# Acoustic Sensing as a Novel Wearable Approach for Cardiac Monitoring at the Wrist

**DOI:** 10.1038/s41598-019-55599-5

**Published:** 2019-12-27

**Authors:** Piyush Sharma, Syed Anas Imtiaz, Esther Rodriguez-Villegas

**Affiliations:** 1 Department of Electrical and Electronic Engineering, Imperial College London, London, UK; 2 Acurable Limited, Cranwood Street, London, UK

## Abstract

This paper introduces the concept of using acoustic sensing over the radial artery to extract cardiac parameters for continuous vital sign monitoring. It proposes a novel measurement principle that allows detection of the heart sounds together with the pulse wave, an attribute not possible with existing photoplethysmography (PPG)-based methods for monitoring at the wrist. The validity of the proposed principle is demonstrated using a new miniature, battery-operated wearable device to sense the acoustic signals and a novel algorithm to extract the heart rate from these signals. The algorithm utilizes the power spectral analysis of the acoustic pulse signal to detect the S1 sounds and additionally, the K-means method to remove motion artifacts for an accurate heartbeat detection. It has been validated on a dataset consisting of 12 subjects with a data length of 6 hours. The results demonstrate an accuracy of 98.78%, mean absolute error of 0.28 bpm, limits of agreement between −1.68 and 1.69 bpm, and a correlation coefficient of 0.998 with reference to a state-of-the-art PPG-based commercial device. The results in this proof of concept study demonstrate the potential of this new sensing modality to be used as an alternative, or to complement existing methods, for continuous monitoring of heart rate at the wrist.

## Introduction

In recent years, the possibility of using wearable monitoring devices as medical devices has been the subject of significant interest worldwide. This is mainly as a result of the potential they could have for early diagnosis of diseases and/or continuous assessment of an individual’s health. Miniaturization of certain types of sensors and electronic interfaces, allows for these to be worn on the body without disturbing the wearer’s daily routine activities. This, consequently makes possible in principle to monitor certain physiological signals as well as physical activity, in some cases over extended periods of time^1^. However, unlike the bulky medical equipment used in clinical settings, wearable electronic medical devices have to be small, light-weighed, low maintenance, easy to handle, and process the recorded data either in the device itself, or wirelessly transmit it to nearby base stations. From the maintenance perspective, the battery life is an important usability constraint, since it is not ideal from a user’s point of view to have to change the batteries frequently. Because of this, the power budget specification of a wearable device is quite important.

Cardiovascular diseases (CVDs) are the number one cause of deaths globally. The number of deaths caused by these diseases are expected to rise with an increase in the average age of the world’s population^2^. However, it is well known that diagnosis and proper follow up and management from an early stage can prevent a large number of such mortalities. It has been shown that continuous monitoring of certain physiological parameters, such as heart rate (HR), blood pressure, cardiac output, pulse wave velocity, etc. can assist in both, an early identification as well as subsequent monitoring of CVDs^3^. This is one of the reasons why, wearable sensors to monitor cardiac parameters, including HR are becoming increasingly popular and a number of non-medical wearable systems have been proposed to monitor cardiovascular parameters and to provide indications of potential cardiovascular diseases. Although wearables have been proposed to be attached in a variety of locations on the body, a majority of these devices operate on the wrist due to the ease of attachment, comfort, aesthetics and adaptability. A major problem of existing wearables, however, is their lack of reliability, i.e. it is difficult to continuously obtain a non-corrupted signal related to cardiac performance using a wearable device^4^. Different approaches have been proposed for HR monitoring.

Electrocardiography (ECG) is the gold standard method for detecting cardiac activity, and obtaining parameters such as HR. ECG is based on sensing the electrical activity of the heart. A typical ECG Holter (i.e. ambulatory) recording setup, consists of a portable recorder and a set of electrodes which are attached to the chest of the subject. Although this setup does not completely constrain the person’s motion, it does limit the daily activities if intended to be used for long term monitoring. These limitations are addressed by some systems, both commercial and proposed in academic papers, that can be used to monitor ECG, with constraints, at different locations on the body. While wearable devices such as the Apple watch (Apple Inc., California, United States), KardiaBand (AliveCor Inc., California, United States), Salutron (Salutron Inc., California, United States), and BioWatch^5^ provide spot measurements of ECG at the wrist, they are not suitable for a long-term cardiac monitoring. Systems such as Zephyr Biomodule (Medtronic Inc., Maryland, United States) and Kenzen patch (Kenzen Inc., California, United States) record the ECG data continuously, however they are worn on the chest and not on the wrist. While Zephyr is widely used in the sports context to measure a number of physiological and biomechanical measurements, Kenzen is currently field testing its technology to monitor the HR on a continuous basis. The electrical activity at different parts of the body can also be sensed by bio-impedance measurements^6,7^, but the use of multiple electrodes, cumbersome wires and large equipment in the recording setup limit its use to hospital wards and intensive care units.

From a usability point of view, a better way of monitoring the cardiac output is based on photoplethysmography (PPG) sensing. PPG is an optics based technique which provides a way of extracting HR by sensing beat-to-beat volumetric changes in arterial blood flow. An extensive amount of commercial PPG-based wearable monitors, mainly smart watches produced by Fitbit (Fitbit Inc., California, United States), Apple (Apple Inc., California, United States), TomTom (TomTom N.V., Amsterdam, Netherlands), Scosche (Scosche Industries Inc., California, United States), etc., allow for continuous HR measurement at the wrist^8–10^. However, the accuracy and reliability of these devices is vulnerable to a number of factors, including motion artifacts, brightness of the environment, or having a stable contact force between the sensor and the measurement site^4^. In addition, PPG conventionally uses an infra-red light of wavelength around 940 nm as an active input signal. This conditions the size of the device, and consequently the length of monitoring, as a result of the power demands of infrared LEDs.

Academic papers have shown how cardiac activity could also be measured using non-contact techniques^11,12^ such as radars^13,14^ and resonators^15^, but these systems are still in very early development stages. Cardiac activity could, in principle, also be measured by using piezoelectric probes^16^, however, these sensors require a stable and continuous pressure through externally applied forces, and are highly sensitive to movements; all of this resulting in a very low signal-to-noise ratio (SNR)^17^.

While all these techniques provide useful information to extract the HR, they suffer from several issues, particularly with the constraints in terms of device size and shape, power budget for a long-term continuous monitoring, reliability and accuracy concerns posed by wearable technologies. Acoustic sensing of chest sounds, using a stethoscope, is the most widely used technique to detect the cardiac output and diagnose heart problems. Acoustic sensing has also been used for other applications^18–20^. As with the sounds on the chest, in this paper we prove that sensing the cardiac rhythms from the radial artery on the wrist is also possible using a very small, low power microphone, without requiring any additional power consuming input signal. This could potentially be used either as an alternative new physiological signal to extract the cardiac information from a wearable device, or as an additional physiological channel to complement existing systems, without posing an overhead in terms of size.

This paper shows for the first time how the HR can be obtained from the acoustic signal sensed with a wearable device attached to the wrist. More specifically, the wrist sensed acoustic signal and its components are characterized using the time-frequency analysis. The optimum position of the sensor in terms of SNR is also investigated. In addition, a novel algorithm to obtain HR, by detecting the peaks corresponding to S1 sounds from the acoustic pulse signal is also proposed. The algorithm utilizes the K-means method to remove artifacts from the signal whereas the PSD estimation allows the extraction of S1 sounds. The results were validated experimentally with human subjects, using standard assessment metrics. The contributions of the presented results are summarized in a discussion, and an insight into the potential of acoustic sensing for monitoring different physiological parameters is finally presented.

## Results

### Acoustic signal characteristics

**Figure 1:**
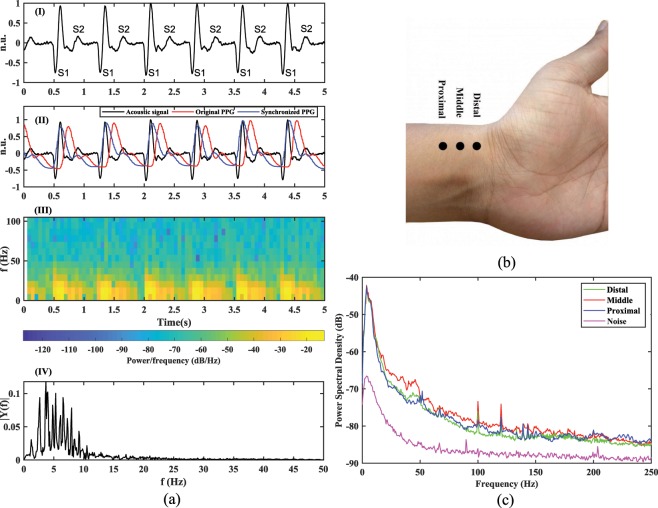
(**a**) Characterization of the acoustic pulse signal: (I) Pulse waveform recorded by placing the miniaturized device designed for this study on the middle position of the radial artery at wrist. (II) Comparison of acoustic and PPG pulse waveforms to synchronize both the signals by matching the nearest systolic peaks. PPG data was recorded using SOMNOscreen pulse oximeter^23^. (III) Joint time-frequency analysis of the acoustic signal obtained using STFT. The color intensity of the grids demonstrates their relative power. (IV) Frequency response (FFT) of the acoustic signal; (**b**) Proximal, middle and distal positions on the radial artery; (**c**) PSDs of the acoustic signal obtained with the microphone placed on distal, middle and proximal site. For illustration, the PSD of the noise was obtained from the signal recorded by completely blocking the microphone port.

Periodic contractions and expansions of the heart muscles generate pressure waves to flow through the arterial system, in systolic and diastolic phases respectively. It is these pressure waves that causes pulse in the arterial system. In the systolic phase, the flow of the blood in the vessel expands the arterial diameter (vasodilatation) whereas a reduction in the arterial diameter (vasoconstriction) is observed in the diastolic phase. These periodic changes in the arterial diameter are transferred through a thin layer of soft tissues and muscles to produce vibrations at the surface of the skin, which can be sensed to understand the dynamics of the heart and the blood vessel wall itself  ^21^. The radial artery is an ideal site for pulse assessment, due to the fact that its vascular properties are less affected by ageing and blood pressure than other arteries in the central region^22^. An example of a pulse waveform recorded by placing a microphone on the radial artery is shown in Fig. 1a(I). It can be seen how the signal mainly consists of two peaks with some intermediate ripples. These are caused, amongst others, by noise of the measuring electronics, electromagnetic interference, and environmental noise. In order to characterize this acoustic signal, the PPG waveform was simultaneously recorded by placing a pulse oximeter^23^ on the index finger. As anticipated, a slight time delay between the onset of the pulse at the radial artery and the index finger was observed. This time delay is a function of the pulse wave velocity and the arterial length. The time delay was empirically found to be nearly constant over the length of the recordings. The synchronization of the acoustic and the PPG pulse waveforms was achieved by overlapping the nearest peaks by removing the time delay, as shown in Fig. 1a(II). It can be observed that the systolic and the diastolic peaks of the PPG signal and the acoustic signal are temporally correlated. Therefore, to resemble with the heart sounds terminology, we term the two peaks in the acoustic signal as S1 and S2 sounds respectively. The frequency response of the acoustic signal, sampled at 2100 Hz, was obtained using the Fast-Fourier transform (FFT). It can be observed that the frequency content of the signal in Fig. 1a(IV) mainly lies below 25 Hz whereas the bandwidth of the heart sounds for a normal subject lie between 20 and 150 Hz^3^. This is because of the high frequency attenuation in the pulse wave caused from the source of the sounds (i.e. the heart) to the measurement site (i.e. the radial artery)^24^. In order to understand the power distribution among different components of the signal, joint time-frequency analysis using short-time Fourier transform (STFT) was performed. The STFT of the signal was obtained using a Blackman window of 256 samples and 50% overlap between consecutive frames. The Blackman window was chosen because it allows a steeper roll-off around the boundaries. The power density of the time-frequency grids in Fig. 1a(III) demonstrates, as expected, that the signal power is mainly concentrated in the S1 and S2 sounds, with S2 sounds carrying a relatively lower energy. However, the signal power and its signal-to-noise ratio (SNR) is also dependent on the sensor location at the wrist.

To determine the optimal auscultation site on the radial artery, the pulse was located at three distinct positions: distal, middle and proximal. The middle position can be easily located in front of the radial styloid process (protruded bone near the wrist crease)^21^. The proximal and distal positions are 1–2 cm on either sides of the middle position, towards the elbow and the wrist crease respectively; as shown in Fig. 1b. A total of nine acoustic recordings, three from each location for every subject, were recorded from a total of 10 subjects to analyze the power spectrum at the different auscultation sites. Note that, although these recordings would be affected by the characteristics of the recording setup, in the experiment the environmental noise and motion artifacts were kept to a minimum. The PSDs of the three recordings for every location, and for every subject were averaged to compare the SNR on the different auscultation sites. For illustration, the PSD of the signal obtained by completely blocking the microphone port is also plotted in Fig. 1c. The latter is an indication of the noise inherent to the sensing system itself in absence of any other sounds. A close correlation between the power spectrum of the signals at different locations can be observed. The anatomy of the radial artery suggests that the vessel depth at the middle position is relatively lower than in the other two sites^25^. Therefore, the operations of vasoconstriction and vasodilatation produces skin surface vibrations with higher amplitudes in the middle location due to a lower attenuation by the surrounding tissues and muscles. This, in turn, results in a higher SNR. The same reasoning can be followed to compare the PSDs of the distal and proximal positions. Due to the ease of locating the middle position, and the insignificant difference between the PSDs, the remaining of the study recorded the acoustic signal with the microphone port facing the middle position of the radial artery.

### HR algorithm’s performance analysis

**Figure 2:**
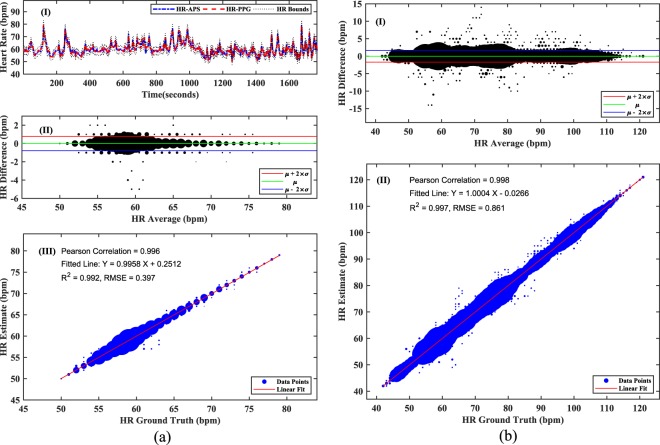
(**a**) Results obtained for one of the subjects: (I) HR comparison between the estimated output (HR-APS) and reference output (HR-PPG) with upper and lower HR bounds of ±5% respectively. (II) Bland-Altman analysis with more than 95% of HR differences lying within LOAs, defined by $(\mu \pm 2\times \sigma )$. (III) Line of best fit between the estimated and ground truth HR values. The *R*^2^ and RMSE value, a measure of fitness of line to the data, were 0.992 and 0.397 respectively. The Pearson correlation was 0.996; (**b**) Results obtained for the complete dataset: (I) Bland-Altman analysis of the HR comparisons for all the subjects. (II) Line of best fit between the estimated and ground truth HR values for all the subjects. The *R*^2^ and RMSE value were 0.997 and 0.861 respectively. The Pearson correlation was 0.998.

In addition to proving the feasibility of obtaining the cardiac signal from the wrist, this study also investigated the possibility of automatically extracting the most fundamental biomarker, namely HR, from the acoustic signal. Since this is the first time such a signal has been sensed via means of wearable acoustic sensing, a novel algorithm had to be created for this purpose. In order to assess the performance of the proposed method, the algorithm results were compared with other state-of-the-art PPG-based devices, for a total of 12 subjects. The ground truth HR values (HR-PPG) were obtained using the FDA approved, and clinically used SOMNOscreen system^23^. The novel algorithm specifically designed to obtain the output from the sensed acoustic pulse signal (APS) provided the estimated HR values (HR-APS). As an illustration, the estimated and ground truth HR values corresponding to 6 recordings, each of 5 minutes duration for one of the subjects, are plotted simultaneously with upper and lower bounds of 5% respectively with respect to HR-PPG, in Fig. 2a(I). The first computed performance metric, shown in Fig. 2a(II), was the Bland-Altman plot^26^. This served to compare the difference between the estimated and ground truth HR values with respect to their corresponding mean. The circled data points in Fig. 2a(II) indicate the HR differences at different HR averages and their diameter corresponds to the number of points coinciding on the same location. The bias *μ* was calculated by averaging all the HR differences, whereas the limits of agreement (LOA) were obtained by computing $(\mu \pm 2\times \sigma )$ respectively, where *σ* is the standard deviation of the HR differences. The bias for this comparison was found to be nearly zero; and LOA indicated a variation of less than 1 bpm for more than 95% of the data points. As a second performance metric, the line of best fit between the estimated and ground truth HR values was also determined, to understand the degree of similarity using Pearson correlation. The *R*^2^ and root-mean-square-error (RMSE) values depict the corresponding measures of fitness of line to the data. A higher value of *R*^2^ and a lower value of RMSE represents a better fit. For the scatter plot in Fig. 2a(III), the fitted line with equation: *y* = 0.9958*x* + 0.2512 was obtained, where *x* indicates the ground truth HR value, and *y* indicates the associated estimate. The Pearson correlation was found to be 0.996 with corresponding *R*^2^ and RMSE values of 0.992 and 0.397 respectively.

A similar analysis was repeated for the complete dataset of 12 subjects, where a total of 6 recordings, each of 5 minutes duration were recorded from every subject. The Bland-Altman comparison and the line of best fit thus obtained are plotted in Fig. 2b. A near zero bias and LOA of [−1.68, 1.69] bpm suggests a narrow difference between the estimated and ground truth HR values over the whole database. The Pearson correlation was approximated to 0.998 with an equation for the line of best fit as: *y* = 1.0004*x* − 0.0266. The corresponding *R*^2^ and RMSE values were 0.997 and 0.861 respectively.

A further evaluation of the proposed method was obtained by computing the mean absolute error (MAE) and the mean absolute error percentage (MAEP) as defined in Eqs. (1) and (2) respectively, where ${{\rm{HR}}}_{est}(i)$ is the estimated HR from the acoustic pulse signal and ${{\rm{HR}}}_{true}(i)$ is the ground truth HR from the SOMNOscreen monitor at the *i*^th^ index in a total of *N* values. MAE as an evaluation index provides an estimate of the deviation across the whole dataset whereas MAEP indicates the percentage of error in the HR estimation. Along with these performance metrics, the standard deviation (*σ*) and Pearson correlation (PC) were also determined to understand the degree of agreement between the corresponding HR outputs. The accuracy of the method was evaluated by calculating the percentage of HR values obtained from the acoustic pulse signal and lying within ±5% of the SOMNOscreen output.

1 $${\rm{MAE}}=\frac{1}{N}\,\mathop{\sum }\limits_{i=1}^{N}\,|{{\rm{HR}}}_{est}(i)-{{\rm{HR}}}_{true}(i)|$$

2 $${\rm{MAEP}}=\frac{1}{N}\,\mathop{\sum }\limits_{i=1}^{N}\,\frac{|{{\rm{HR}}}_{est}(i)-{{\rm{HR}}}_{true}(i)|}{{{\rm{HR}}}_{true}(i)}\times 100$$

**Figure 3:**
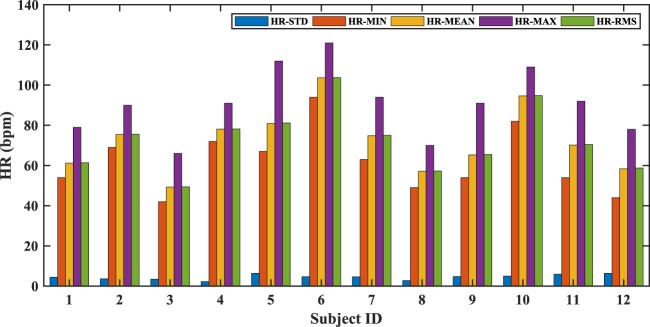
Variation of HR in individual subjects. HR-STD: standard deviation of the range; HR-MIN: minimum value of the range; HR-MEAN: mean value of the range; HR-MAX: maximum value of the range; HR-RMS: root-mean-square value of the range.

Table 1 lists the performance metrics of the proposed method for all of the 12 subjects. An overall accuracy of 98.78% with a mean absolute error and a standard deviation of 0.28 and 0.86 bpm respectively, were obtained. Figure 3 plots the HR variations in individual subjects including the standard deviation (HR-STD), minimum (HR-MIN), mean (HR-MEAN), maximum (HR-MAX) and root-mean-square (HR-RMS) of the corresponding HR range. The HR in the complete dataset varied from 42 to 121 bpm.

**Table 1:** Performance metrics of the proposed method obtained by comparing the estimated and ground truth HR.

	P01	P02	P03	P04	P05	P06	P07	P08	P09	P10	P11	P12	Total
MAE (bpm)	0.10	0.34	0.19	0.24	0.36	0.14	0.18	0.15	0.40	0.34	0.38	0.61	0.28
MAEP (%)	0.17	0.44	0.42	0.31	0.44	0.13	0.24	0.26	0.62	0.36	0.53	1.03	0.39
*μ* (bpm)	−0.01	−0.18	0.03	0.03	−0.01	0.01	−0.01	−0.02	0.01	0.09	0.07	−0.06	0.01
*σ* (bpm)	0.39	1.19	0.47	0.66	0.84	0.38	0.49	0.85	0.96	0.90	1.17	1.49	0.86
LOA (bpm)	[−0.78, 0.77]	[−2.48, 2.12]	[−0.88, 0.94]	[−1.26, 1.32]	[−1.65, 1.64]	[−0.73, 0.75]	[−0.98, 0.96]	[−1.68, 1.63]	[−1.87, 1.90]	[−1.67, 1.85]	[−2.23, 2.38]	[−2.97, 2.86]	[−1.68, 1.69]
PC	0.996	0.948	0.991	0.956	0.991	0.997	0.994	0.953	0.979	0.983	0.980	0.971	0.998
Acc (%)	99.91	96.92	99.74	99.01	98.75	99.87	97.29	98.41	97.73	99.19	98.14	94.05	98.78

The proposed method was also tested using acoustic signals recorded in a noisy environment. The signals of 5 minutes duration were collected from 5 subjects. During the experiment, the subjects were asked to read a page of text and loud music was played in background at the same time. The results in Table 2 indicate that the effect of environmental noise on the acoustic pulse recordings for the HR determination are insignificant.

**Table 2:** Performance metrics of the proposed method for acoustic signals recorded in a noisy environment.

	P01	P02	P03	P04	P05
MAE (bpm)	0.26	0.20	0.36	0.63	0.09
MAEP (%)	0.41	0.28	0.47	0.89	0.14
*μ* (bpm)	−0.08	−0.07	−0.11	0.06	0.03
*σ* (bpm)	0.69	0.48	0.89	1.99	0.35
LOA (bpm)	[−1.45, 1.28]	[−1.02, 0.88]	[−1.87, 1.64]	[−3.83, 3.96]	[−0.65, 0.72]
PC	0.970	0.988	0.936	0.861	0.986
Acc (%)	99.29	98.12	98.81	95.00	99.05

Table 3 compares the results of the proposed method with other studies which analyzed the accuracy and reliability of different state-of-the-art PPG-based wrist devices used in the commercial market by comparing them with the synchronous ECG signal. Although these devices were tested under different experimental conditions such as sitting in rest position, walking, and running at different speeds and slopes, Table 3 only includes the results corresponding to the data recorded at the rest position to provide an indicative comparison with the proposed method. Note that to the best of the authors’ knowledge, there is no database or any other study which has published results on HR monitoring using an acoustic pulse signal, and therefore a direct comparison could not be established. Also, the devices in these studies were tested on different number of subjects, but the total data length were quite similar to this study. The table follows the same abbreviations for the comparison parameters as used in the literature. The mean error (ME) and standard deviation (SD) of the HR differences have the same definitions as *μ* and *σ* respectively. These parameters obtain a value of 0.01 bpm and 0.86 bpm for the proposed method and are significantly lower than other devices. The MAE and MAEP in this work are found to be 0.28 bpm and 0.39%, and demonstrates better performance in comparison to the devices analyzed by Stahl *et al*.^8^ and Parak *et al*.^27^. A higher PC of 0.99 as compared to 0.96 for Basis Peak and 0.83 for Fitbit Charge HR, as studied by Jo *et al*.^10^, also indicates a higher agreement between the estimated and ground truth HR for the proposed method. The standard error (SE) of the mean measures the deviation in the mean HR of all the subjects and attains a higher value of 4.55 bpm in this study. This is mainly because the SE is inversely proportional to the square root of the sample size^28^. Since the other studies were tested on a higher number of subjects, the inverse proportionality results in a lower estimate of the SE. The comparison over these parameters show that, considering PPG is a widely accepted technique, the proposed method utilizing the acoustic sensing can provide accurate results for HR monitoring at wrist under equivalent conditions.

**Table 3:** Performance comparison of the proposed method with results obtained from different PPG-based wrist devices used in the commercial market.

Literature	Wearable Device	Subjects	Data^+^ Length	ME (bpm)	SD (bpm)	MAE (bpm)	MAEP (%)	PC	SE (bpm)
Stahl *et al*.^8^	Scosche Rhythm	50	5.0 hr	—	1.64*	—	2.22	—	1.60
	Mio Alpha			—	1.52*	—	2.72	—	1.50
	Fitbit Charge HR			—	1.45*	—	7.73	—	1.40
	Basis Peak			—	1.58*	—	3.15	—	1.50
	Microsoft Band			—	1.52*	—	3.81	—	1.40
	TomTom Runner Cardio			—	2.06*	—	2.54	—	2.00
Parak *et al*.^27^	Mio Alpha	21	4.2 hr	−0.20	—	3.92	5.37	—	—
	Scosche Rhythm			0.07	—	4.83	5.96	—	—
Jo *et al*.^10^	Basis Peak	24	6.0 hr	−0.20	—	—	—	0.96	6.04
	Fitbit Charge HR			−3.73	—	—	—	0.83	10.66
Cadmus *et al*.^9^	Basis Peak	40	6.7 hr	2.75	9.93	—	—	—	—
	Fitbit Charge			−0.65	4.92	—	—	—	—
	Fitbit Surge			−0.30	2.40	—	—	—	—
	Mio Fuse			1.05	4.42	—	—	—	—
Spierer *et al*.^39^	Omron HR500U	47	4.7 hr	2.22^†^	—	—	—	—	3.67^†^
	Mio Alpha			2.39^†^	—	—	—	—	6.28^†^
This Work	Proposed Acoustic Device	12	6.0 hr	0.01	0.86	0.28	0.39	0.99	4.55

The table only compares the results of the data collected at the rest position and provides an illustrative comparison because the experimental conditions varied between different works. ^+^The data length is for all the subjects combined together. *SD was calculated from the results of 95% equivalence testing given in this paper. ^†^The results provided in the paper were obtained by averaging the data to 5 seconds epochs.

## Discussion

The feasibility of acoustic sensing of the radial pulse using a wearable device has been investigated in this paper. While ECG has always been used as the gold standard method to record cardiac signals from the chest, measuring it continuously with a wearable device presents lots of limitations, varying from reliability to usability. An alternative to ECG, which improves on the usability aspects, is to use PPG-based devices instead. This approach is very popular due to the fact that it allows monitoring with the sensor attached on the wrist. But methods based on wrist PPG are not limitations free either. The requirements of an active input signal limit either the size of the system and/or the battery lifetime. In addition the systems are very sensitive to motion and other artefacts. Hence having an alternative lower power sensing approach would be desirable to either complement the PPG to increase the sensing accuracy, or replace it altogether, depending on the clinical target. The passive sensing mechanism of state-of-the-art acoustic sensors (MEMS microphones) imposes significantly less constraints in terms of power, hence being more suitable from the size and maintenance perspective for a wearable device.

In this work, the optimal auscultation site on the radial artery was also studied, since this is a factor to consider when comparing the ease of sensor attachment with respect to ECG- and PPG-based approaches. It was proven that acoustic sensing allows for a relatively wide region of sensor placement with an insignificant difference between the SNR of the signals recorded from different locations over the radial artery.

The characteristics of the pulse wave originating from the heart- as a result of the opening and closing of the heart valves, and propagating as a mechanical wave along the arterial branches were also investigated, by comparing the acoustic and PPG pulse waveforms. Although negligible, the heart sounds also transmit an acoustic wave through the body^14^. Since these acoustic features are superimposed on the vessel vibrations caused by the mechanical constriction and dilation of the radial artery, a similar type of skin surface modulation is obtained. While PPG only measures the pulse wave component, it was proven how acoustic-based sensing allowed the detection of both cardiophysiological characteristics of the radial pulse. This was done by observing the bandwidth of the acoustic pulse waveform, which contained energies in the audible range as compared to bandwidth of less than 10 Hz for the PPG waveform^29^. Consequently, with the proposed approach it is shown that it is possible to monitor both, the heart sounds as well as the pulse wave using just one wearable system. These findings could be utilized in the future work to, for example, study different phases of the Korotkoff sounds at the wrist, to measure blood pressure using a wearable device.

Furthermore, by comparing the HR obtained from acoustic sensing with other state-of-the-art PPG based devices, it was shown that the presence of fundamental heart sounds in the acoustic pulse waveform improved the heartbeat detection, an important variable in continuous vital sign monitoring. Heartbeat detection based on extraction of S1 sounds using the new proposed method further reduced the error between the estimated and ground truth HR and achieved a high accuracy of 98.78% with a PC of 0.99 and narrower LOAs of [−1.68, 1.69] bpm. These results prove that the proposed method could be used as an alternative, or to complement PPG for continuous monitoring of HR at wrist. It is worth noting, however, that although the proposed method for HR has been tested experimentally, this paper presents just the proof of concept. To be used as part of a medical device, full clinical validation would require testing on a larger cohort wearing a device based on this principle in an ambulatory setting. This would allow not only to investigate a wider range of cardiac signals, but also to test with real life artifacts.

As a summary, with this work, we showed for the first time, that the acoustic signal sensed from the radial artery in the wrist can be used as a novel physiological signal to extract biomarkers indicative of cardiac performance. Furthermore, this signal provides advantages with respect to other conventionally used ones, which make it specially suitable for wearable devices. The concept and feasibility has been proven with the automatic extraction of HR. In future work, automatic extraction of other cardiac biomarkers could be investigated, such as HR variability using the inter-beat intervals, pulse transit time, pulse wave velocity, etc.

## Methods

### Acoustic sensing

**Figure 4:**
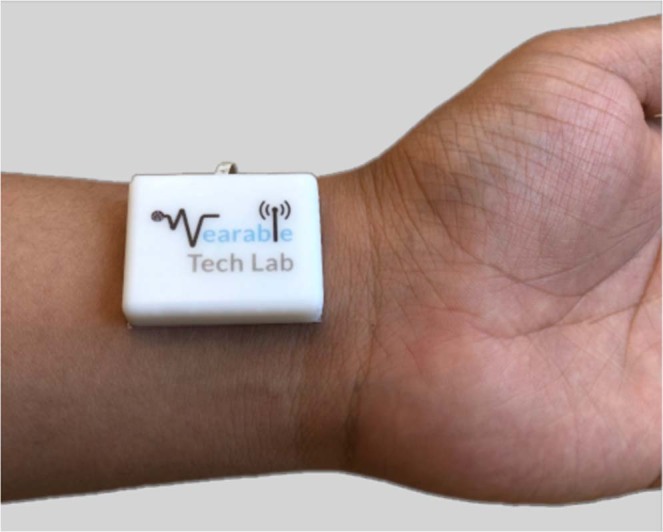
Wearable device used to acquire acoustic signals. The device consists of a MEMS microphone sensor integrated with Bluetooth low energy transmission, and powered by a 3.7 V coin cell battery (20 mm in diameter).

The periodic pumping of the blood through the cardiovascular system in the body generates dilation and constriction cycles in the radial artery. As a result, periodic variations in the arterial diameter occur which produce corresponding vibrations at the surface of the skin. These vibrations introduce changes in the surrounding air pressure which can be transferred to the diaphragm of a suitable microphone. Long term monitoring of these vibrations using a miniaturized device requires a sensor with a small form-factor, operating with very low currents so that the whole system can run with a small battery over a suitably long period of time. For this study, we designed a miniature, battery-operated wireless device as shown in Fig. 4 using an ultra-low noise, omnidirectional MEMS microphone sensor (InvenSense INMP411). The MEMS microphone was chosen because MEMS technology offers excellent acoustic characteristics with very small form factors. This is achieved through a fabrication process which involves creating a moveable membrane and a fixed backplate over a cavity in the base silicon wafer^30–32^. While the perforations in the fixed backplate allows air to flow easily through it, the moveable membrane flexes in response to the change in surrounding air pressure caused by sound waves. These movements change the capacitance between the backplate and the membrane, which can be sensed by an application specific integrated circuit to convert the vibro-acoustic effects in an electrical signal. The chosen microphone has a high SNR of 62 dBA, a uniform sensitivity of −46 dBV between 28 Hz and 20 KHz, and a low power consumption of 210 uA at 3.3 V supply^33^. However, any microphone of similar size and specifications could be used. The analogue output of the microphone after appropriate filtering and amplification was digitised using an inbuilt analogue-to-digital converter of a Nordic Semiconductor nRF52 Series chip. This chip also contained a Bluetooth low energy transceiver for the wireless transmission of the data using a 2.4 GHz chip antenna (Johanson Technology Inc.). The overall weight of the final wireless prototype was 8 grams, although note that this could be further optimized by using more sophisticated manufacturing processes. In addition, its size and shape were designed so that it could be easily attached to the wrist using double sided medical adhesive tapes to keep the sensor affix to the measuring site, for a long-term usage^34^.

### Algorithmic blocks

**Figure 5:**
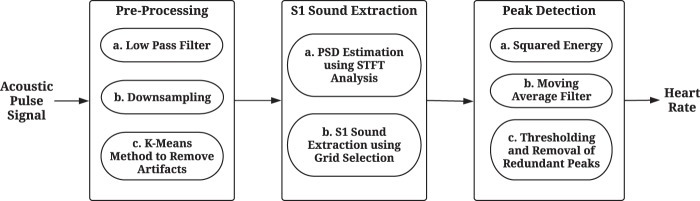
Block diagram of the proposed algorithm to determine HR from the acoustic signal by extracting S1 sounds using the STFT analysis.

An overview of the novel algorithm proposed to automatically determine the HR by extracting the S1 sounds from the acoustic pulse signal is shown in Fig. 5. The algorithm mainly consists of 3 stages: 1- The pre-processing blocks reduce contamination of the signal caused by noisy artifacts, in order to improve the SNR for further analysis; 2- The PSD of the signal is calculated in the following stage using STFT to extract the S1 sounds; 3- Finally, the peaks corresponding to these sounds are detected to provide a time index by constructing a squared energy envelope for HR determination. A pseudo-code for the proposed algorithm is also provided in Table 4. The following sections explain the details of the different blocks.

**Table 4:** Pseudo-code algorithm for estimating HR from acoustic pulse signal. The symbol notations are referenced in the main text.

1. Initial pre-processing of the signal.	2. S1 sound extraction from acoustic pulse signal.
• Acoustic pulse signal: **y**, sampled at *f*_*s*_ = 2100 Hz.	• Joint time-frequency analysis: **PSD** = STFT(y).
• Low-pass filtering: **LPF**(y), with *w*_*c*_ = 25 Hz.	• Maximum power, **P**_**max**_ = max(PSD).
• Downsampling operation: ↓**10**(y), with *f*_*d*_ = 210 Hz.	• Extract grids with **P** ≥ P_max_ − P_t_, where ${{\rm{P}}}_{{\rm{t}}}\in [5,10]\,{\rm{dB}}$ such that ${\rm{m}}\in [4,17]$.
• K-means method: Form two clusters by scoring the signal parts y_n_ using **S**_**n**_ = {0, 1} for ${\rm{n}}\in [1,5]$.	• Identify S1 regions: (**t**_**sa**_ − 0.15, **t**_**ea**_ + 0.15), ${\rm{a}}\in [1,{\rm{m}}]$.
3. Peak detection from extracted S1 sounds.	4. Find the continuous average HR.
• Squared energy: **y**^**2**^.	• Find the time indexes for maximum of energy peaks: **T**_**m**_ = max(${E}_{m}$).
• Averaging filter: ${\int }_{{\bf{1}}}^{{\bf{32}}}\,{{\bf{y}}}^{{\bf{2}}}$.	• Estimate the HR: ${\bf{H}}{\bf{R}}=\tfrac{60}{({\sum }_{m=1}^{4}\,\Delta {T}_{m})/4}$
• Artifact elimination using thresholds: **W**_**z**_ and **A**_**z**_.	

#### Acoustic data pre-processing

The acoustic signal sensed at the wrist contains not just the signal of interest but also other signals that are picked up by the electronic system, such as motion artifacts and sounds from the surrounding environment. In order to achieve a better SNR by reducing the effects of the latter, the acoustic signal, denoted by time-series *y* is processed into rectangular windows of 5 seconds duration with 1 second of overlap between successive segments. The window length is chosen to include enough number of heart beats corresponding to an HR in a range of 40 to 200 beats per minute (bpm).

Most of the frequency content of the acoustic signal is contained below 25 Hz. Because of this, undesired higher frequency interference/noise is reduced by using a fifth-order Butterworth low-pass filter with a cut-off frequency of 25 Hz. The acoustic signal originally sampled at 2100 Hz (*f*_*s*_) possesses frequencies well below the corresponding Nyquist frequency after the filtering process. This redundant information is therefore removed by downsampling the signal by a factor of 10 reducing the sampling frequency to 210 Hz (*f*_*d*_), without introducing any aliasing in the signal.

Since the signals were continuously recorded in a session of 30 minutes duration, the subjects were allowed to move their wrist or fingers during the data acquisition. These movements could possibly introduce acoustic vibrations which can be sensed by the microphone and introduce large amplitudes in the signal. The frequencies corresponding to these artifacts can lie within the bandwidth of the acoustic pulse signal, which would not be eliminated with simple low-pass filtering. However, the effect of such movements usually lies in smaller time frames. Because of this, K-means clustering method^35^ with two classes, *C1* and *C2*, is used in the algorithm to identify the parts of the signal which are significantly corrupted by them. The method initially divides the signal blocks, *y*, of 5 seconds duration, into five equal parts, each of 1 second duration, and denoted by *y*_*n*_, $n\in [1,5]$. For every part, the maximum amplitude (*A*_*max*_) and the standard deviation (*σ*) are determined to reflect the signal characteristics as *x* and *y*-coordinates respectively. These feature coordinates are fed to the K-means blocks to cluster the five signal parts into two different classes based on the similarity of the features. The method proceeds by choosing two cluster centroids, *O1* and *O2*, and groups the features into two classes by iteratively updating the centroid coordinates, $({x}_{O1},{y}_{O1})$ and $({x}_{O2},{y}_{O2})$, to minimize the feature points-to-cluster-centroid distances. Once the iterative process converges, the horizontal change, Δ*x*, between the centroids is determined, and the class *C* with a lower standard deviation is found. A change of less than 50% in Δ*x* reflects a close correspondence between the maximum amplitudes of different signal parts, and indicates no significant corruption by the motion artifacts. Since the artifacts exhibit a higher standard deviation than the acoustic pulse signal, the class with a lower *y*-coordinate is chosen in cases where the change in Δ*x* is more than 50%. Depending on the comparison between these parameters, in Eq. (3), the signal parts *y*_*n*_ are scored by assigning *S*_*n*_, $n\in [1,5]$ a value of either 1 or 0. The signal parts with a score of 1 are ignored from the further processing.

3 $$\begin{array}{rcl}\Delta x & = & \frac{|{x}_{O1}-{x}_{O2}|}{{\min }({x}_{O1},{x}_{O2})}\\ C & = & \{\begin{array}{ll}C1, & {\rm{if}}\,{y}_{O1}\le {y}_{O2}\\ C2, & {\rm{if}}\,{y}_{O1} > {y}_{O2}\end{array}\\ \mathop{{S}_{n}}\limits_{n\in [1,2,3,4,5]} & = & \{\begin{array}{ll}\{\begin{array}{lll}1 & \forall  & n\in C2\\ 0 & \forall  & n\in C1\end{array} & {\rm{if}}\,\Delta x\ge 0.5\,\& \,C=C1\\ \{\begin{array}{lll}1 & \forall  & n\in C1\\ 0 & \forall  & n\in C2\end{array} & {\rm{if}}\,\Delta x\ge 0.5\,\& \,C=C2\\ \{0 & {\rm{if}}\,\Delta x < 0.5\end{array}\end{array}$$

**Figure 6:**
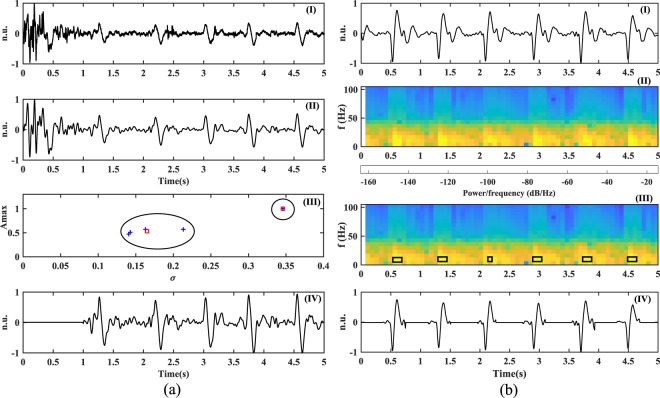
(**a**) Pre-processing of the acoustic signal sensed by the system: (I) Original signal. (II) Low-pass filtered and downsampled signal to remove higher frequency components and redundant information respectively. (III) Clustering using the K-means method to identify signal segments corrupted with motion artifacts. Symbol + and □ represents the features and cluster centroids respectively. (IV) Signal segment corrupted with motion artifact (due to wrist/finger movement) removed from the downsampled signal; (**b**) S1 sounds extraction from a different pre-processed signal with no corrupted segment: (I) Acoustic signal after initial low-pass filtering, downsampling and K-means application. (II) PSD of the signal obtained using STFT to extract S1 sounds. (III) Rectangular windows representing the regions of interest. (IV) S1 sounds extracted by adding a tolerance of 150 milliseconds on both sides of the rectangular windows.

Figure 6a shows different pre-processing stages for a 5 seconds block of signal, a part of which is significantly corrupted by motion artifacts. It can be seen how the processing results on successfully ignoring the first part of the signal from the further processing.

#### S1 sound extraction

An HR in a range of 40 to 200 bpm corresponds to a beat-to-beat interval of 1500 to 300 milliseconds respectively. The number of S1 sounds in a 5 seconds window therefore can vary from 4 to 17. The measured PSD of the acoustic pulse signal showed that the frequencies corresponding to the S1 sounds, in the joint time-frequency analysis, carried higher power than other parts of the signal. This property of the signal is utilized to extract these sounds in the time-domain and process them further to find the HR. But it was also important to select a proper window length for calculating the PSD of the signal, as a better time resolution allows the extraction of the S1 waveform without interfering much with the nearby signal transitions.

The power spectrum of the acoustic pulse signal with a downsampled frequency of 210 Hz is calculated in the algorithm using a Blackman window of 32 samples (approximately 150 milliseconds) with an overlap of 50% between successive frames. The chosen time window, as shown in Fig. 6b(II), provides the required time resolution to extract the S1 waveform by segmenting the time axis into a relatively higher number of grids. The colour intensity of these grids in the time-frequency space indicates their corresponding contribution to the overall power of the signal. The grid with the maximum power, *P*_*max*_ is found and all the grids with power not differing more than 5 dB with respect to *P*_*max*_ are also selected. It is understood that the beat-to-beat interval cannot be lower than 300 milliseconds^3^ therefore, all the grids with a mutual separation within this time period supposedly belong to a single S1 sound, and hence they are all grouped together, as shown by rectangular windows in Fig. 6b(III). For *m* of such groupings, the starting and end time points, *t*_*sa*_ and *t*_*ea*_, where $a\in [1,m]$, are noted. The threshold difference of 5 dB (*P*_*t*_) is increased in steps of 1 dB, up to a maximum of 10 dB, to limit these *m* number of groupings for a 5 seconds window between 4 and 17. A tolerance window of 150 milliseconds, observed empirically, is added to *t*_*sa*_ and *t*_*ea*_ to enlarge the region of interest in the time-domain, and ensure that the S1 waveform is completely extracted. Only the signal corresponding to the group timings of $({t}_{sa}-0.15,{t}_{ea}+0.15)$ seconds is retained, whereas the other parts of the signal are zeroed for the further processing as shown in Fig. 6b(IV).

#### Peak detection

**Figure 7:**
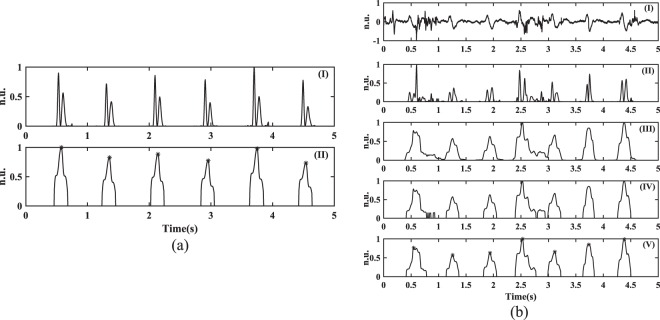
(**a**) Peak detection in a clean signal: (I) Squared energy of the S1 sound waveform in Fig. 6b(IV). (II) Energy peaks obtained using the moving average filter. $\ast$ Represent the time indexes corresponding to the S1 sounds; (**b**) Peak detection in a corrupted signal: (I) Input acoustic signal corrupted with motion artifacts (introduced by wrist/finger movements). (II) Squared energy of the signal obtained after PSD analysis. The redundant peaks due to the motion artifacts in systolic and diastolic phases of the cardiac cycle can be observed. (III) Energy envelope obtained using the moving average filter. (IV) Thresholding of energy peaks to remove envelopes corresponding to the motion artifacts. (V) Time indexes of energy peaks corresponding to S1 sound waveforms in the signal. This shows how the algorithm successfully distinguishes between motion artifacts and S1 waveforms.

Constructing energy envelope of the extracted S1 sounds. Although a number of peak detection methods using the joint time-frequency analysis exist^36,37^, the power spectrum of the acoustic signal obtained using STFT provides an easy way to detect the S1 sounds as the peaks. However, it is important to determine a single time-index for every S1 sound in the signal, so that their mutual time differences can be utilized to calculate the HR. To obtain the peak-indexes, every sample of the signal is first squared so that the positive and the negative waveform of the S1 sounds can be transformed to only positive amplitudes above the baseline as shown in Fig. 7a(I). The squaring process provides a nonlinear amplification of the signal by emphasizing the higher frequencies corresponding to the S1 sounds, whilst attenuating the nearby transitions with lower energies.

A moving average filter is subsequently used to integrate the squared energy waveform. The width of the integration window is an important parameter to consider and should ideally be equal to the maximum time duration of the S1 sound in the signal. A window with a larger width can combine the energy of the S1 sound with the energy of nearby signal transitions, whereas a narrower window can produce multiple energy envelopes for the same sound^38^. For a signal with a sampling frequency of 210 samples/second, the filter averages the squared energy waveform over a window of 32 samples. The squared energy followed by an averaging process therefore produces an energy peak corresponding to the S1 sound, as shown in Fig. 7a(II) which can be easily processed to find the corresponding time index.

Artifact identification and elimination. In the pre-processing stage of the proposed algorithm, there were some instances when the artifacts introduced by the wrist or finger movements significantly corrupt some sections of the acoustic signal and were not detected by the K-means method. This happened when the maximum amplitude and standard deviation of the signal corrupted with artifacts were close to the features of cleaner sections in a 5 seconds window. Since these artifacts may have significant power, in comparison to the S1 sounds, the STFT analysis allows such signal transitions to appear as well in the further analysis. The energy envelopes of such sections corrupted with artifacts could introduce misleading energy peaks affecting the accurate determination of time indexes. To avoid the misclassification of an artifact as the S1 sound, features such as time width and amplitude of every energy peak, are determined in the algorithm. For the acoustic signal, the total number of 5 seconds blocks is defined as *L*, where ${y}_{z}[n]$, $z\in [1,L]$ represents each signal block. Assuming that the parameter ${l}_{z}$ provides the total count of energy peaks in ${y}_{z}[n]$, the width and amplitude features of every energy peak ${E}_{m}$ are denoted by ${w}_{m}$ and ${a}_{m}$, respectively, where $1\le m\le {l}_{z}$. The thresholds ${W}_{z}$ and ${A}_{z}$ to process the segment under consideration are determined using Eqs. (4) and (5) respectively, by computing the average of the time widths and amplitudes of all the energy peaks present in the last three signal blocks. The initial value of these thresholds are determined by processing the first six data blocks (30 seconds of the signal) and analyzing the corresponding features of the energy peaks.

4 $${W}_{z}=\frac{{\sum }_{m=1}^{{l}_{z-1}}\,{w}_{m}+{\sum }_{m=1}^{{l}_{z-2}}\,{w}_{m}+{\sum }_{m=1}^{{l}_{z-3}}\,{w}_{m}}{{l}_{z-1}+{l}_{z-2}+{l}_{z-3}}$$

5 $${A}_{z}=\frac{{\sum }_{m=1}^{{l}_{z-1}}\,{a}_{m}+{\sum }_{m=1}^{{l}_{z-2}}\,{a}_{m}+{\sum }_{m=1}^{{l}_{z-3}}\,{a}_{m}}{{l}_{z-1}+{l}_{z-2}+{l}_{z-3}}$$

Since the characteristics of the energy peaks corresponding to the S1 waveforms are continuously computed, the thresholds automatically adapt to the changing behaviour of the data, i.e. are not static in value. With the thresholds ${W}_{z}$ and ${A}_{z}$ calculated for the segment under consideration, the following criteria filter out the energy peaks from the further processing:

1. The energy peaks ${E}_{m}$ in Fig. 7b(III) are clipped using an amplitude threshold equal to (0.25 × *A*_*z*_). All the data points above this threshold are retained, while rest of the envelope is zeroed.
2. The thresholding procedure produces redundant peaks as shown in Fig. 7b(IV) which should be filtered out to avoid an incorrect determination of time indexes. The width ${W}_{z}$ evaluated for the current segment is utilized in Eq. (6) to remove the unnecessary peaks. The resultant energy peaks thus obtained correspond to the S1 sounds in the signal.
  6 $$\begin{array}{lll} &  & \,\forall \,m\in [1,{l}_{z}]\\ {E}_{m} & = & \{\begin{array}{ll}{E}_{m}, & {\rm{if}}\,0.75\times {W}_{z}\le {w}_{m}\le 1.25\times {W}_{z}\\ 0, & otherwise\end{array}\end{array}$$
3. Finally, all the time indexes (also referred as HR indexes) corresponding to the maximum of the energy peaks, as indicated by $\ast$ in Fig. 7b(V), are noted. These time locations and the number of energy peaks after the artifact removal procedure are defined as ${T}_{m}$ (in seconds) and ${t}_{z}$ respectively, where $1\le m\le {t}_{z}$.

The time indexes obtained after processing the signal block under consideration can be utilized to determine the beat-to-beat interval, Δ*T* in Eq. (7). The HR is calculated every ${(1/4)}^{th}$ second by averaging the beat-to-beat time intervals corresponding to the last 4 heart beats and multiplying it by 60 as follows:

7 $$\begin{array}{l}\forall \,m\in [1,{t}_{z}-1]\\ \begin{array}{rcl}\Delta {T}_{m} & = & {T}_{m+1}-{T}_{m}\\ {\rm{HR}} & = & \frac{60}{({\sum }_{m=1}^{4}\,\Delta {T}_{m})/4}\end{array}\end{array}$$

### Subjects and experimental protocol

Signals were recorded from 12 healthy subjects aged 19–48 by placing the new miniature, battery-operated wearable device over the radial artery. The sensor attachment, over an area equal to the size of the sensor (27 × 20 millimetres), did not require any cleaning process. The data was recorded only through contact sensing without applying any external pressure on the device. The signals were sampled at a frequency of 2100 Hz and wirelessly transmitted to a nearby base station. The PPG signals from the index finger were simultaneously recorded using a commercially available SOMNOscreen pulse oximeter^23^. The SOMNOscreen monitor also provided an estimate of the HR every ${(1/4)}^{th}$ second. The monitor uses a methodology to determine the HR for which the details are not publicly available. A total of 6 recordings, each of 5 minutes duration were recorded from every subject. All the recordings were collected in an uncontrolled environment, but the subjects were asked to sit and relax on a chair. Since the recordings were performed for a long duration, the subjects could move their wrist and fingers, as and when required. The synchronization of the data from both the sensors, which is critical to evaluate the performance of the proposed system, was carried out by matching the nearest systolic peaks.

### Human subjects

The study was approved by the local ethics committee of Imperial College London (ICREC reference number: 18IC4358). All research was performed in accordance with relevant guidelines and regulations. The informed consent was obtained from all the subjects in human trials.

## Data Availability

The data that support the findings of this study are available from the corresponding author upon reasonable request.
